# Development of Citrus-Based Functional Jelly and an Investigation of Its Anti-Obesity and Antioxidant Properties

**DOI:** 10.3390/antiox11122418

**Published:** 2022-12-07

**Authors:** Mingfang Peng, Zhipeng Gao, Yanfang Liao, Jiajing Guo, Yang Shan

**Affiliations:** 1 Longping Branch, College of Biology, Hunan University, Changsha 410125, China; 2 International Joint Lab on Fruits & Vegetables Processing, Quality and Safety, Hunan Key Lab of Fruits & Vegetables Storage, Processing, Quality and Safety, Hunan Agriculture Product Processing Institute, Hunan Academy of Agricultural Sciences, Changsha 410125, China; 3 College of Animal Science and Technology, Hunan Agricultural University, Changsha 410128, China

**Keywords:** *chenpi*, antioxidant, orange juice, anti-obesity, jelly, functional food

## Abstract

Intervention with natural products is becoming a promising obesity control strategy as healthy eating becomes increasingly popular. The present study aimed to prepare a citrus-based functional jelly (CFJ) from citrus by-products and investigate its bioactive effects in mice. The results of the CFJ preparation showed that the optimal formula of CFJ was 29.12%, 20%, and 3.61% for *chenpi*, orange juice, and pectin, respectively. The optimized CFJ can be personalized and designed with jelly shapes using 3D food printing technology. The evaluation of the biological activity of the CFJ showed that it was low in calories, with a total phenolic content of 12.44 ± 0.26 mg GAE/g. Moreover, the CFJ has a good free radical scavenging ability for ABTS. The results of the mouse experiments showed that the CFJ significantly suppressed the body weight gain and fat deposits with a dose-dependent effect, compared with the control group (*p* < 0.05). In addition, the activities of the antioxidant-related enzymes (CAT and SOD) of the mice were also enhanced after a supplementation with the CFJ. In short, the CFJ is a functional snack enriched in phenolic substances with low-calorie, antioxidant and anti-obesity properties. This work promotes the utilization of citrus by-products and the healthy development of its processing industry.

## 1. Introduction

With the abundance of resources and the rapid development of society, human dietary habits and diet structure have also changed. The long-term excessive intake of a high-calorie diet has become an essential cause of the high prevalence of metabolic chronic conditions, such as obesity, nonalcoholic fatty liver, hyperlipidemia, and diabetes [1,2,3]. Thus, dietary intervention and prevention are emerging as a potential treatment strategy for obesity and its metabolic syndrome [4]. The increasing amount of research has found that obesity is tightly linked to oxidative stress [5,6]. The consumption of a high-calorie diet promotes oxidative stress, not only by inducing the mitochondrial dysfunction and peroxisomal oxidation of fatty acids, but also by reducing the activity of antioxidant enzymes, such as superoxide dismutase (SOD) and catalase (CAT) [7]. Therefore, dietary supplementation with antioxidants will contribute to weight management and obesity prevention [8,9]. In addition, dietary fiber could control the appetite by regulating the gastrointestinal transport [10], and the fermentation of products, such as short-chain fatty acids, in the intestine, which also improve metabolic diseases by regulating the gut microbiota [11]. The development of safe, effective, and long-term obesity prevention products has become a hot research topic, especially the development of products, based on natural resources.

Citrus is one of the most abundant types of fruit in the world, and its juice is widely consumed because of its flavour and beneficial properties [12]. Orange (*Citrus junos Sieb. ex Tanaka*) is one of the most common fruits for fruit juice processing. Its juice is rich in vitamin C and phenolic compounds [13], especially flavonoids, which have been proven to have anti-adipogenesis and anti-obesity activities [14]. At the same time, citrus derivatives have been widely studied for their numerous health benefits. *Chenpi* is made from the dried and aged peel of *Citrus reticulata Blanco* and its cultivated variants in the *Rutaceae* family. In the homologous culture of medicine and food, *chenpi* occupies a critical position [15]. When used as traditional Chinese medicine (TCM), *Chenpi* is most frequently used to regulate Qi and strengthen the spleen [15]. Qi could be understood as the energy circulating throughout the body [15]. To regulate Qi in TCM, refers to the use of medicine to treat Qi-stagnation, Qi-flowback or Qi-deficiency [16], which in layperson’s terms means soothing our emotions, including anger, irritability, and frustration. *Chenpi* is rich in various bioactive components, including volatile oils, flavonoids, alkaloids, and polysaccharides [17]. It has been increasingly found that *chenpi* could exert anti-inflammatory and antioxidant effects [18,19], by regulating the blood glucose homeostasis [20] and prevent obesity [18]. In addition, pectin extracted from citrus peel residue is a soluble dietary fiber and is frequently used as a dietary supplement. Pectin brings many health benefits by regulating the gut flora, such as modulating the immune barrier in the gastrointestinal tract and controlling or preventing inflammatory conditions [21,22]. Generally, pectin can be classified into high methoxylated pectin (HMP) and low methoxylated pectin (LMP), and the latter is commonly used as an essential material for the making of jelly [23]. As the main by-product of citrus processing, applying pectin in the citrus peel to jelly production will boost the efficient utilization of citrus resources.

People tend to prefer healthy and leisurely foods, in modern society. As mentioned above, orange juice, *chenpi*, and LMP all show favorable advantages in promoting health benefits. Therefore, we aimed to develop a formulation for functional jelly, based on citrus products. A single-factor experiment (SFE) and response surface methodology (RSM) were used to optimize the formulation of citrus-based functional jelly (CFJ). Moreover, the 3D food printing technology was performed to further explore the potential of a personalized design of the CFJ. Further, the bioactivities of the CFJ were explored in vitro and in vivo. We expect that this work can provide a new idea for the future functional food industry, while providing consumers with more food consumption options.

## 2. Materials and Methods

### 2.1. Materials

Oranges (navel orange) were purchased from a local store in Changsha and produced in Hunan Province (Changsha, China). The *chenpi* (red *Pericarpium Citri Reticulatae*) that was aged for 3 years, was bought from the Jiangmen Xinhui Tangerine Peel Market Co., Ltd. (Jiangmen, China). LMP, extracted from citrus peel (purity > 98%), was obtained from Xi’an Shouherb Biotech Co., Ltd. (Xi’an, China). Food-grade CaCl_2_ was purchased from Chongqing Ruihancheng Technology Co., Ltd. (Chongqing, China). All ingredients used in the preparation of the jelly were food-grade.

### 2.2. Development of the Citrus-Based Functional Jelly

#### 2.2.1. Preparation of the Citrus-Based Functional Jelly

Prior to the preparation of the citrus-based functional jelly, the *chenpi* is crushed, to obtain *chenpi* powder. The decoction method of traditional Chinese medicine was used, 20 g of *chenpi* powder was added to 400 mL of drinkable water, decocted for 40 min, and then filtered through a vacuum filter while hot. The decoction of *chenpi* was concentrated to a concentration of 1 g/mL, using a rotary evaporator (N-1300V-W, Tokyo Rikakikal Co., Ltd., Tokyo, Japan) at 50 °C and 0.09 MPa. The orange was peeled and squeezed, to obtain orange juice. The jelly samples were prepared, according to the previous studies reported [24,25]. Briefly, LMP was added to 20 mL of drinkable water and stirred in a water bath at 65–75 °C, until the pectin was completely dissolved, after which different volumes of the *chenpi* decoction and orange juice were added and mixed well. Then, 10 mL of the CaCl_2_ solution of different concentrations was added, and an appropriate amount of drinkable water was added to bring the total volume to 100 mL. Finally, the mixture was homogenized and canned into conventional jelly moulds, pasteurized, and stored at 4 °C.

#### 2.2.2. Sensory Test of the Citrus-Based Functional Jelly

The sensory evaluation is generally considered a scientific product evaluation method for analysing people’s satisfaction with a product through their senses of sight, smell, touch, and taste. The analysis of the CFJ’s sensory evaluation was carried out by a sensory assessment panel consisting of 20 people trained in food science. The four aspects of the CFJ, colour and lustre, texture, aroma, and taste, were evaluated and scored. The scores of the above four components were added together to obtain the total’ sensory evaluation score of the CFJ, and the optimal formulation showed the highest total sensory score. The sensory scoring criteria are shown in Table 1.

#### 2.2.3. Design of the Single-Factor Experiments (SFEs)

The SFE was used to evaluate the influence of adding different amounts of the *chenpi* decoction, orange juice, pectin, and CaCl_2_, on the sensory evaluation of the CFJ. It can provide a reference for the factor levels in the response surface optimization experiments. The calcium ions are necessary for preparing the jelly using LMP, which forms a gel state with pectin. The concentration range of the CaCl_2_ in the CFJ was selected from 0.8–18 mg/mL, with reference to the results reported in a previous study [26]. The content of the *chenpi* decoction, the main functional ingredient of the CFJ, was selected in the range of 10–35%, based on the recommended daily intake of *chenpi* for humans [15]. The orange juice content was chosen as 15–40%, as orange juice could enhance the citrus aroma of the CFJ and increase the vitamin C content of the product. Pectin is the essential ingredient for the preparation of the jelly, which provides a toughness while keeping the shape of the jelly. Thus, 1–6% of the pectin content in the CFJ was selected with reference to Peng M.F, et al. [27]. The jelly samples were produced by mixing different concentrations of the *chenpi* decoction, orange juice, pectin, and the CaCl_2_ base, on the procedure described. Each CFJ sample was sensorily evaluated and the sensory assessment panel gave a total sensory evaluation score.

#### 2.2.4. Design of the Response Surface Methodology (RSM)

The RSM is a statistical method that uses a rational experimental design and experiments to investigate the functional relationship between the factors and the response values to seek the optimal process parameters [28]. The levels of each factor of the RSM were picked from the findings of the SFE. Following the SFE experiments, the optimal calcium addition concentration was obtained, we further investigated the effects of the three main functional components of the CFJ on the total sensory evaluation of the CFJ, using the RSM experiments. In the subsequent experiments, the concentration of CaCl_2_ in all of the CFJ formulations was maintained at 1.4 mg/mL. A three-factor and three-level experiment were designed using the Box–Behnken model. The effects of the *chenpi* decoction, orange juice, and the pectin content in the CFJ on the total sensory evaluation, were explored. The independent variables A, B, and C were the addition of the *chenpi* decoction, orange juice, and citrus pectin, and their three levels are denoted by 1, 0, and −1, respectively. The detailed factors and the levels of the RSM are shown in Table 2. The optimal formulation of the CFJ was determined through a regression equation using the total sensory evaluation score as the response value (R). The best formulation for the preparation of the CFJ was used as a sample for the subsequent experiments.

#### 2.2.5. 3D Food Printing of the Citrus-Based Functional Jelly

The objective of this experiment is to investigate whether the CFJ, optimized by the formulation, can be used for the personalized jelly shape design using 3D food printing. The CFJ samples are prepared using the best formula obtained from the RSM experiments and then used as raw material for the 3D printing. The CFJ 3D printing trials were completed in a professional 3D food printing company (Hangzhou Shiyin Technology Co., Ltd., Hangzhou, China). A 3D food printer (Foodbot S2, Hangzhou Shiyin Technology Co., Ltd.) with a nozzle diameter of 0.84 mm was used to conduct the printing experiments. The precise print shape of the CFJ (cube: 15 × 15 × 15 mm) was preset using Cura15.02.1 software (Ultimaker BV, Arnhem, Netherlands). The printing infill velocity (mm/s) and the infill density (%) defines the deposition rate of the material and the amount of material extruded the during printing, both of which are key factors in determining the success of the 3D printing [29]. Two different printing parameters were set, according to the producer’s printing recommendations: the infill velocity of 10 (mm/s) with an infill density of 100% and an infill velocity of 15 (mm/s) with an infill density of 90%. In addition, the printed infill pattern was set to be linear and the printed temperature was 40 °C. The optimal printing parameters were determined, based on the appearance and size of the finished CFJ print, to match the design structure.

### 2.3. Assay of the Main Nutritional and Bioactive Contents of the Citrus-Based Functional Jelly

The measurement indicators for the content of the main nutritional composition in the CFJ include energy, protein, fat, carbohydrate, and sodium. The above components were measured or calculated according to their respective Chinese national standards [27].

The concentration of the total flavonoids (TFs) in the CFJ, was assayed by a spectrophotometric method, as previously published [30]. In brief, 0.5 mL of the CFJ solution was mixed with 12.5 mL of 30% ethanol and 1 mL of 5% sodium nitrite solution, and incubated for 6 min. Then, the mixture was mixed with 1 mL of a 10% aluminium nitrate solution. Then, following 6 min of the reaction, 10 mL of a 4% sodium hydroxide solution was mixed. The absorbance at 510 nm was measured within 15 min, using a spectrophotometer (Shimadzu Instrument Co., Ltd., Suzhou, China). The content of the TF is expressed as mg rutin equivalent (RE)/g fresh weight.

The concentration of the total phenolics (TP) was determined using the Folin– Ciocâlteu reagent [31,32]. In brief, 20 µL of the CFJ solution was mixed with 9.98 mL distilled water and 1 mL of the Folin–Ciocâlteu reagent. Then, after avoiding a light reaction for 5 min, 5 mL of 5% aqueous sodium bicarbonate and 9 mL of distilled water were added, then mixed and placed in the dark for 60 min. The absorbance of the mixture was determined at 765 nm using a spectrophotometer (Shimadzu Instrument Co., Ltd., Suzhou, China). The content of the TP is represented as mg gallic acid equivalent (GAE)/g fresh weight.

The specific phenolics and flavonoids contained in the CFJ were analysed using HPLC. The chromatographic column was a Sustain C_18_ 250 mm × 4.6 mm column (particle size 5 μm; Shimadzu, Kyoto, Japan). The column temperature was 30 °C, the mobile phase A1 was 0.1% formic acid water, mobile phase was B-methanol, and the flow rate was 0.25 mL/min. The gradient elution conditions were 0–1 min, 10% B; 1–3 min, 10–33% B; 3–10 min, 33% B; 10–15 min, 33–50% B; 15–20 min, 50–90% B; 20–21 min, 90% B; 21–22 min, 90–10% B; 22–25 min, 10% B. The injection volume was 5 μL. All substances in the CFJ were quantified using the standard curves for the respective substances.

### 2.4. Assessment of the Antioxidant Ability of the Citrus-Based Functional Jelly In Vitro

The antioxidant ability of the CFJ was measured with three commonly used methods, namely 2,2-azinobis (3-ethyl-benzothiazoline-6-sulfonic acid) (ABTS) and 2,2-diphenyl-1-octanohydrazide (DPPH) radical scavenging methods, and the ferric reducing antioxidant power (FRAP) methods [33]. Trolox, a commonly used antioxidant, was used as a positive control experiment, and the antioxidant capacity of the CFJ was also expressed as µmol Trolox equivalents (TEs)/g fresh weight. Since the antioxidant capacity of the CFJ itself exceeds the assay threshold in the kit, the sample needs to be diluted. In our experiments, the CFJ for the ABTS scavenging ability test was diluted 80-fold, the CFJ for the DPPH scavenging ability test and the FRAP reduction ability identification was diluted 50-fold with the extraction buffer in the kit. Finally, these assays were based on the instructions of the corresponding kits, purchased from Suzhou Comin Biotechnology Co., Ltd. (Suzhou, China). In brief, 950 µL of the ABTS reaction solution was added to 50 µL of the sample, shaken for 10 min, and the absorbance was measured at 734 nm using the microplate reader (Thermo Scientific, Waltham, MA, USA). Simultaneously, 950 µL of the DPPH reaction solution was added to 50 µL of the sample, shaken for 20 min, and the absorbance was measured at 515 nm by the microplate reader. Meanwhile, 190 µL of the FRAP reaction solution was added to 10 µL of the sample, shaken for 20 min, and the absorbance was measured at 593 nm by the microplate reader. The antioxidant activity was calculated using the following equations:

ABTS scavenging activity (µmol Trolox/g) = (A_control_ − A_sample_ − 0.0208)/0.5012/V_sample_ × 80 (1)

DPPH scavenging activity (µmol Trolox/g) = (A_control_ − A_sample_ − 0.0404)/0.6616/V_sample_ × 50 (2)

FRAP reducing capacity (µmol Trolox/g) = (A_sample_ − A_control_ + 0.0062)/1.1164/V_sample_ × 50 (3)

### 2.5. Investigation of the Bioactivities of the Citrus-Based Functional Jelly in Mice

#### 2.5.1. Animal Experiment

Four-week-old male C57BL/6J mice were bought from the Hunan SJA laboratory animal Co., Ltd. (Changsha, China). All animals were kept in an environment with a temperature-controlled (21–23 °C) and light-dark cycle (12 h/12 h). The animals had free access to food and drinking water. All animals were administered and treated in conformity with the protocols approved by the Hunan Agricultural University Institutional Animal Care and Use Committee (202005).

Following seven days of adaptive culture, forty mice were assigned, randomly, to the following four groups (*n* = 10), control group (C), high concentration CFJ group (H), medium concentration CFJ group (M), and low concentration CFJ group (L). Mice in groups H, M, and L were orally gavaged with 10, 5, and 2.5 g/kg body weight (BW), of CFJ, per day, for 8 weeks, respectively. The medium concentration of the CFJ was converted from the maximum recommended daily intake of *chenpi* for humans [15]. Group C was given the equivalent amount of saline, as well. All groups were fed a normal diet (Hunan SJA Laboratory Animal Co., Ltd., Changsha, China) throughout the experiment. The main nutritional composition of the normal diet is shown in the Table 3. The BW and food intake of the mice were measured and recorded every week. Following 8 weeks of experimentation, the blood samples were obtained from the orbital sinus of the mice, and then the mice were killed by cervical dislocation. Liver samples, subcutaneous adipose tissue (SAT) samples, abdominal adipose tissue (AAT) samples, and brown adipose tissue (BAT) samples of the mice were immediately collected and weighed.

#### 2.5.2. Histological Examination

The liver samples were fixed with a 4% paraformaldehyde solution and the SAT was fixed with a fat fixative. The liver and SAT samples were dehydrated with ethanol, embedded with paraffin, sectioned, and stained with hematoxylin and eosin (H&E) [34]. The tissue sections were visualized and photographed using a light microscope (Nikon Eclipse E100, Tokyo, Japan) at 40× magnification.

#### 2.5.3. Serum Chemistry Analysis

The blood samples were centrifuged with a frozen centrifuge at 4 °C, 3000 rpm for 10 min, and the serum supernatant was removed and stored at −80 °C until the assay [35]. The concentrations of the serum total cholesterol (TC), serum triglycerides (TG), serum low-density lipoprotein cholesterol (LDL-C), and serum high-density lipoprotein cholesterol (HDL-C) were assessed. The markers of the antioxidant capacity were measured, covering superoxide dismutase (SOD) and catalase (CAT). All serum indexes were measured following the protocol of the corresponding kit obtained from Nanjing Jiancheng Technology Co., Ltd. (Nanjing, China).

### 2.6. Statistical Analysis

The SPSS software (version 26.0, IBM, New York, USA) was used for the statistical analysis. The data compared between the various groups were analysed using a nonparametric test. All of the data were mapped using GraphPad Prism 8. All results were shown as mean ± standard deviation. A *p* < 0.05 was considered a statistically significant difference.

## 3. Results

### 3.1. Development of the Citrus-Based Functional Jelly

#### 3.1.1. The Effect of the Single Factor on the Citrus-Based Functional Jelly

**Figure 1:**
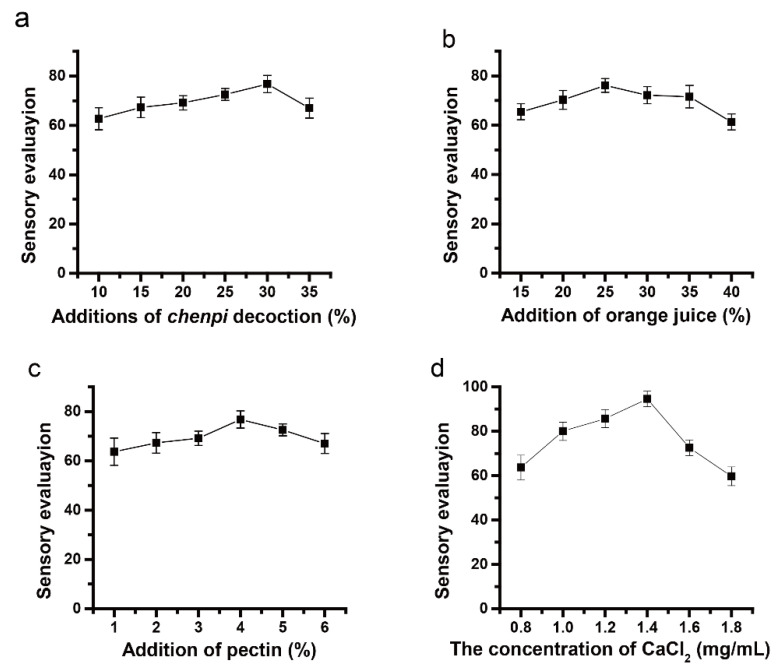
The effect of the single factor on the CFJ. (**a**) The effect of the additions of the *chenpi* decoction on the total sensory evaluation of the CFJ (the highest total sensory score for CFJ at 30% *chenpi* decoction); (**b**) the total sensory evaluation score of the CFJ varies with the additions of orange juice (15%-40%) (the highest total sensory score for CFJ at 25% orange juice); (**c**) effect of pectin on the total sensory evaluation of the CFJ (the highest total sensory score for CFJ at 4% pectin); (**d**) effect of the concentration of CaCL_2_ on the total sensory evaluation of the CFJ (the highest total sensory score for CFJ at 1.4 mg/mL CaCL_2_).

As can be seen from Figure 1a, the total sensory evaluation (TSE) of the CFJ increased with the increase of the *chenpi* decoction, and the TSE reached its highest when the content of the *chenpi* decoction was 30%. Still, after that, the TSE showed a decrease. (Figure 1a). Figure 1b indicates the effect of the orange juice content on the TSE of the CFJ, when the amount of the *chenpi* decoction was 30%. We observed an upward trend in the TSE of orange juice, ranging from 15% to 25%, then decreased with the increase of the orange juice concentration (Figure 1b). It showed that the optimal amount of orange juice to be added was 25%. In addition, the effect of pectin on the TSE of the CFJ when the *chenpi* decoction was added at 30% and the orange juice content was 25%, is illustrated by Figure 1c. The TSE of the CFJ rose with the increased addition of pectin from 1~4%, while the further growth of the pectin addition induced a decline in the TSE. Based on the above single-factor test results, the optimal CaCl_2_ concentration was investigated at a 30% addition of the *chenpi* decoction, a 25% addition of orange juice, and a 4% addition of pectin. The optimal concentration of CaCL_2_ is 1.4 mg/mL, based on the total sensory results (Figure 1d). Jellies formed with the calcium ion concentrations above 1.4 mg/mL, have a poorer taste, resulting in lower scores in the TSE. Thus, the range of 25–35% for the *chenpi* decoction, the range of 20–30% for orange juice, and the range of 3–5% for pectin, were selected for the RSM. The concentration of CaCl_2_ in all CFJ formulations was maintained at 1.4 mg/mL.

#### 3.1.2. Optimization of the Citrus-Based Functional Jelly Preparation Formulations by the RSM

We applied the RSM (Box–Behnken design) to optimize the development of the CFJ. The design of the factors, the levels, and the corresponding results are illustrated in Table 4. The following regression equation of the three factors and the total score of the sensory evaluation (R) was obtained by conducting the result analysis:

R = −307.375 + 23.765 A − 2.5 B + 47.425 C + 0.12 AB − 0.65AC + 0.1 BC − 0.409 A^2^ − 0.059 B^2^ − 4225 C^2^ (4)

The adequacy, reproducibility, and accuracy of the model can be checked with a F-test, *t*-test, and a R^2^ analysis. As shown in Table 5, the total *p*-value of the model was <0.0001, which indicates that the model is valid. The *p*-value of the lack of fit was 0.1523 > 0.05, which suggests that the original hypothesis cannot be rejected and the model can be considered as not misfit. Moreover, the R^2^ of 0.981 for the model showed an agreement with the adjusted R^2^ (0.957), suggesting that the actual R values matched favorably with the predicted values. In addition, the coefficient of variation (CV) of the model was 2.4% (<10%) and the value of adequate precision was 20.6 (>4), indicating a favourable reproducibility and accuracy. Therefore, the response value R can be predicted at different levels of the independent variables from this regression equation.

The ANOVA results showed that both orange juice and pectin had an exceptionally significant effect (*p* < 0.01) on the sensory evaluation (R-value), while the *chenpi* decoction also had a significant effect (*p* < 0.05). The F-value indicates the significance of the influence of the factor on the R-value, with larger F-values indicating a greater importance. In this model, the effect of orange juice on the sensory evaluation of the CFJ was greater than the other two ingredients. The *p*-values in the model indicated that AC, A^2^, and C² were extremely significant differences (*p* < 0.01), AB was significantly different (*p* < 0.05), and the other factors were not significant (*p* > 0.05).

**Figure 2:**
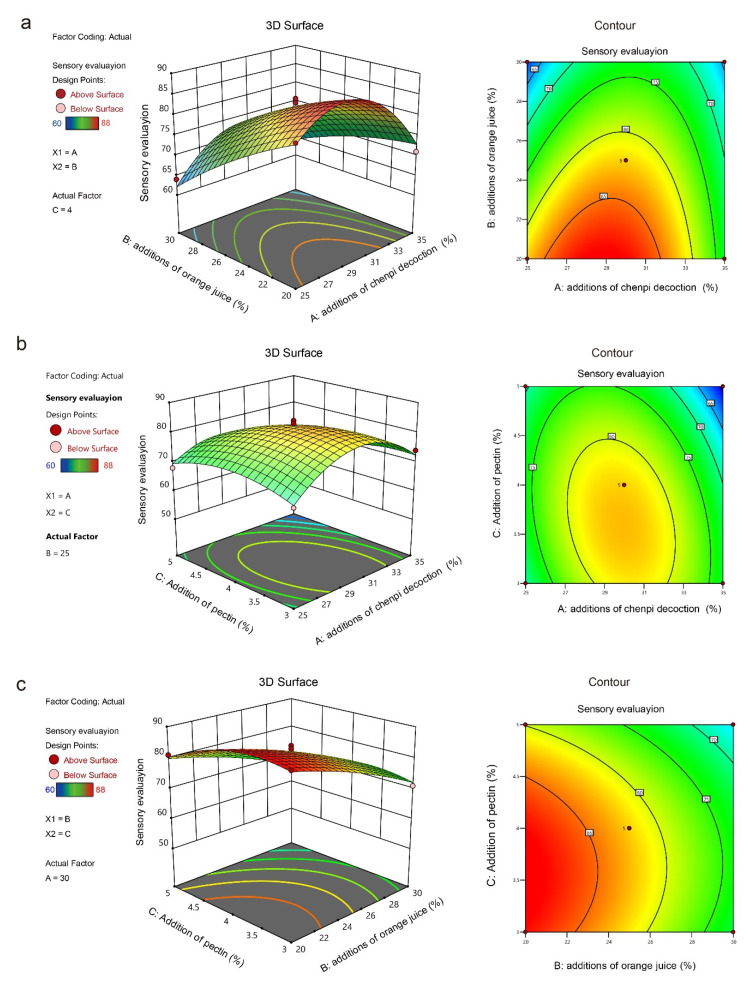
Response surface plots showing the effect of the *chenpi* decoction with orange juice (**a**), *chenpi* decoction with pectin (**b**), and orange juice with pectin (**c**) on the sensory evaluation.

Both the three-dimensional (3D) response surface and the contour plots could represent the interaction relationship between the factors. A steep response surface and elliptical contours indicated a significant interaction between the factors. The contours of AB and AC were nearly elliptical and the 3D response surface was relatively steep, suggesting a remarkable interaction between these two factors (Figure 2a,b). Although the 3D response surface of BC was steep, the contour was similar to circular, showing no significant interaction between the two factors (Figure 2c). The findings are consistent with the ANOVA analysis.

Finally, the optimal predicted value of the response value R was obtained at 29.12% for the *chenpi* decoction, 20% for orange juice, and 3.61% for pectin, with R (predicted) = 89.16, and the relative error between the actual test result (R = 88.23) was only 0.93%, suggesting a relatively high fit of the model.

#### 3.1.3. 3D Food Printing of the Citrus-Based Functional Jelly

**Figure 3:**
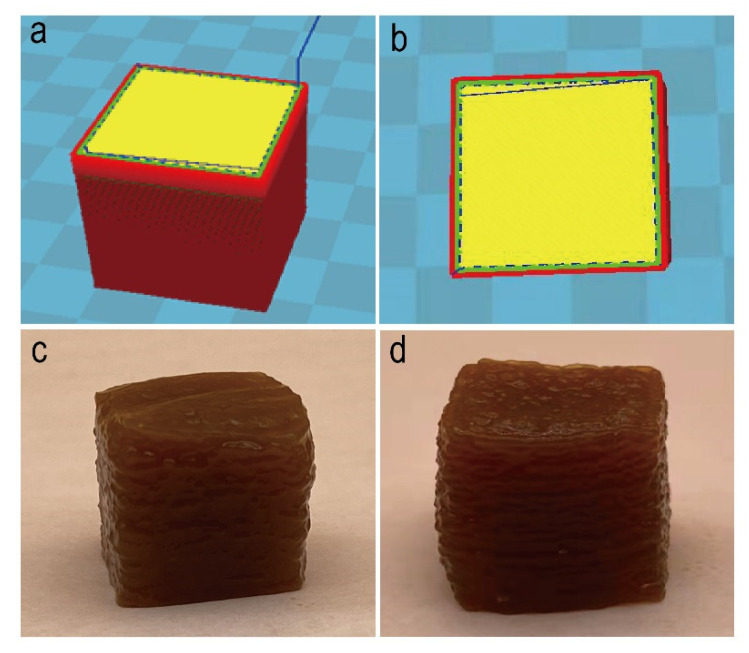
Three-dimensional food printing of the CFJ. (**a**) Three-dimensional printed shape of the 15 × 15 × 15 mm cube, designed with Cura15.02.1 software. (**b**) Cross-sectional view of the 3D shape. The yellow part is the print infill part, and the middle slash indicates the infill patterns of the CFJ. (**c**) Three-dimensional printed cubic jelly obtained with an infill velocity of 10 (mm/s) with an infill density of 100%. (**d**) Three-dimensional printed cubic jelly obtained with an infill velocity of 15 (mm/s) with an infill density of 90%.

The 3D food printing test was performed to evaluate the feasibility of the personalized printing for the CFJ. Both printing parameters set in our tests were generally successful in printing the expected jelly shape. We have pre-designed a desired print shape for the CFJ, as shown in Figure 3a (cube 15 × 15 × 15 mm). The cross-section of the printed shape shows the visualization of the filling direction of the material extrusion during the printing process (Figure 3b). Successfully, we have the finished print (Figure 3c,d). When the printing parameter is the infill velocity of 15 (mm/s) with an infill density of 90%, the jelly obtained has a minor depression in the middle, due to material deposition, by observing the appearance of the jelly and measuring its size. Overall, the printed CFJ sample matched well with the designed structure by measuring the size of the CFJ.

### 3.2. Analysis of the Main Nutritional and Bioactive Contents of the Citrus-Based Functional Jelly

#### 3.2.1. Nutritional Composition

Usually, the products developed are subjected to the determination of their five main ingredient content, including energy, protein, fat, carbohydrate, and sodium. As shown in Table 6, a little protein and fat content was found in the CFJ, 0.1% and 0.53%, respectively. Moreover, the carbohydrate content in the CFJ was 20.4%, and the energy of the CFJ in 100 g fresh weight was calculated to be 360 kJ (86.12 calories).

#### 3.2.2. Determination of the Bioactive Substances and the Antioxidant Capacity of the Citrus-Based Functional Jelly

**Figure 4:**
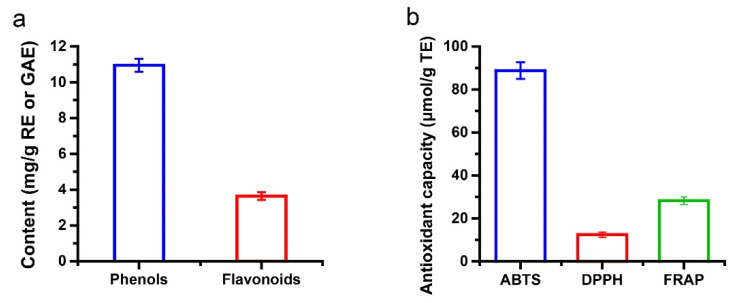
The content of the bioactive components of the CFJ (**a**) and its antioxidant capacity, in vitro (**b**).

The phenols and flavonoids are closely related to the antioxidant capacity of the CFJ. In this part, we measured the content of the TP and TF in the CFJ, respectively, and also determined their antioxidant capacity in vitro. The optimized CFJ contains 12.44 ± 0.26 mg GAE/g fresh weight and 3.64 ± 0.21 mg RE/g fresh weight of the TP and TF, respectively (Figure 4a). Further, the specific phenolics were quantified. The CFJ was detected to contain 22 phenolic compounds, of which the six with the highest concentrations were hesperidin, nobiletin, naringin, diosmin, naringenin, and sinensetin (Table 7). Two of the top six most abundant substances were poly methoxy flavones (nobiletin and sinensetin).

Meanwhile, the antioxidant capacity of the CFJ was measured in vitro. As shown in Figure 4b, the scavenging effectiveness of the CFJ on the ABTS and DPPH radicals was the equivalent of 88.87 ± 3.87 and 12.44 ± 0.26 μmol TE/g fresh weight, and the reducing capacity of the FRAP was 28.24 ± 0.84 μmol TE/g fresh weight.

### 3.3. Bioactive Effects of the Citrus-Based Functional Jelly on Healthy Mice

#### 3.3.1. Citrus-Based Functional Jelly Preventing the Development of Obesity

**Figure 5:**
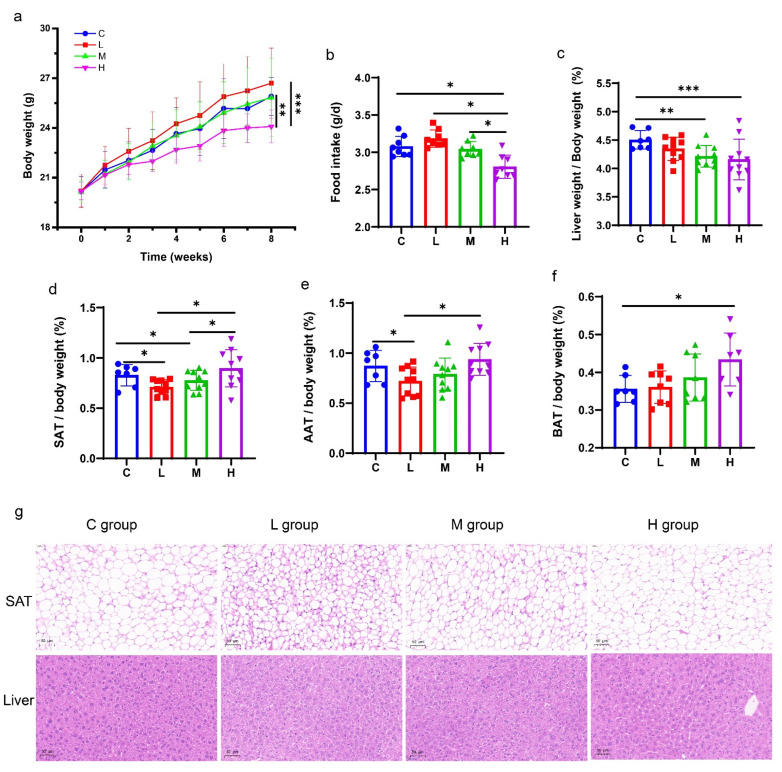
CFJ preventing the development of obesity. (**a**) Body weight changes in mice (*n* = 7–10, the control group was expressed as C, and the groups supplemented with low concentration, medium concentration, and high concentration FCJ were expressed as L, M, and H, respectively), (**b**) the average daily food intake of the mice, the ratio of the liver (**c**), subcutaneous adipose tissues (**d**), abdominal adipose tissues (**e**), and brown adipose tissues to the body weight (**f**), (**g**) The observation of the SATs and liver by H&E staining (40×). * *p* < 0.05, ** *p* < 0.01, and *** *p* < 0.001.

Following 8 weeks of the CFJ supplementation, the body weight of mice decreased extremely, in the high concentration CFJ group (H) compared with the control group (C) (*p* < 0.001) (Figure 5a). The low concentration CFJ group (M) showed a trend of promoting weight gain, but there was no significant difference. The weight of the mice in group C and the medium concentration CFJ group (M) was similar. At the same time, the food intake of the mice supplemented with the CFJ, decreased in a dose-dependent manner, and group H was remarkably lower than group C (*p <* 0.001) (Figure 5b). The ratio of the liver to body weight also decreased in a dose-dependent manner, the groups M and H had significantly lower results than group C (*p* < 0.001 and *p* < 0.001). Interestingly, the body fat rate of the mice increased with the increase of the CFJ supplementary concentration (Figure 5c). Compared with group C, the ratio of the subcutaneous adipose tissues (SATs) to the body weight, decreased significantly in group L and group M (*p* < 0.05), but there was no significant change in group H (Figure 5d). Meanwhile, the ratio of the abdominal adipose tissues (AATs) to the body weight in group L, was significantly lower than that in group C (*p* < 0.05), but there was no significant effect in group M and H (Figure 5e). Notably, we observed a trend of a dose-dependent increase in the brown adipose tissues (BATs) to the body weight ratio in mice following the CFJ supplementation, the H group was raised significantly, compared to the C group (Figure 5f).

Consistently, the histopathological examination of the SATs showed that the inhibitory effects of the CFJ on the adipocyte enlargement, were negatively correlated with the concentrations (Figure 5g). The enlargement of the adipocytes was obviously inhibited in the L group, in comparison with the C group. The M group and H group also showed a particular inhibitory effect, which showed that the cell size of the adipose tissue (SAT) was directly proportional to the body fat rate. There was no difference in the histopathological examination of the liver of mice between the four groups, with an intact liver histology, regular hepatocyte morphology, and no steatosis or inflammation (Figure 5g). These findings indicated that the CFJ could potentially prevent obesity by reducing body weight gain and fat accumulation, and controlling the appetite of the mice.

#### 3.3.2. Impact of the Citrus-Based Functional Jelly Supplementation on the Blood Lipid Profile

**Figure 6:**
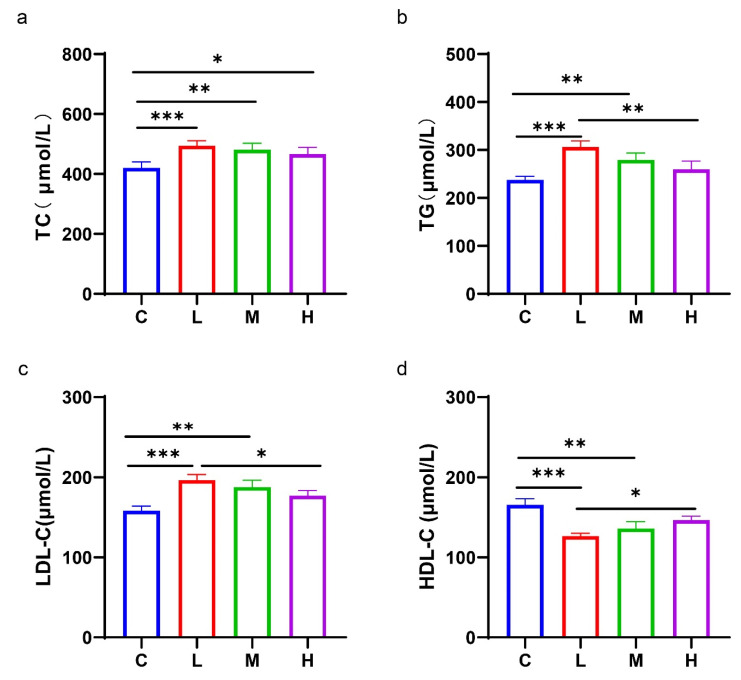
Impact of the CFJ supplementation on the blood lipid profile. Level of cholesterol (TC) (**a**), triglycerides (TG) (**b**), low-density lipoprotein cholesterol (LDL-C) (**c**), high-density lipoprotein cholesterol (HDL-C) (**d**) in serum (*n* = 7–10, since mice have individual differences, the mice had unexpected deaths during rearing that were not related to the experimental material, and in addition, mice that had serious deviations from normal weight during rearing were excluded). * *p* < 0.05; ** *p* < 0.01; *** *p* < 0.001.

The concentration of the TC, TG, and LDL-C in the serum of the mice supplemented with the CFJ, was significantly increased, while the level of the HDL-C was significantly lower, compared with group C (*p* < 0.05) (Figure 6). However, this phenomenon was alleviated with the increase of the supplementary concentration of the CFJ.

#### 3.3.3. Citrus-Based Functional Jelly Enhances the Antioxidant Ability In Vivo

**Figure 7:**
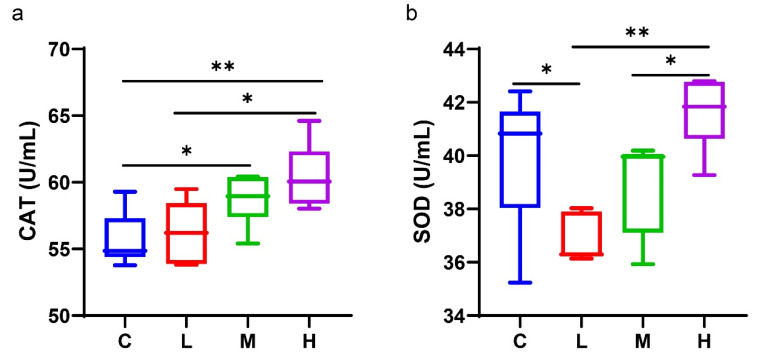
CFJ improved antioxidant ability in vivo. The activity of catalase (CAT) (**a**) and superoxide dismutase (SOD) in serum (**b**). * *p* < 0.05 and ** *p* < 0.01.

The activity of CAT and SOD in the serum reflects the body’s ability to alleviate oxidative stress. As shown in Figure 7, the CAT activity in the serum was significantly increased in groups M and H, compared with group C, whereas there was no significant effect in the L group (Figure 7a). It is noteworthy that the SOD activity was significantly decreased in group L, compared to group C. In contrast, there was no significant difference in other groups (Figure 7b). Although there was no significant effect, we find that the serum antioxidant ability of mice showed an increasing trend after the administration of our product with a dose-dependent effect. These findings indicate that the antioxidant ability of the mice could be enhanced by supplementing with the CFJ.

## 4. Discussion

As lifestyles change and people focus more on eating healthy, many natural products with health benefits are being discovered. Based on the obesity management strategy of natural product intervention, we have developed a functional snack (CFJ) that is well suited to satisfy the demand of people for nutrition and health. In previously published studies, *chenpi* has shown excellent antioxidant effects in vitro and in vivo [36,37]. Therefore, *chenpi* was the main functional ingredient during the development process, which provides the CFJ with more prosperous bioactive substances and better health benefits. In the SFE, we noticed a decrease in the CFJ sensory scores with the increasing content of *chenpi* above 30%. It may be because the bitter compounds, such as limonoids in the *chenpi,* can be accumulated in the CFJ as the concentration of the *chenpi* decoction increases [15]. Orange juice can provide the CFJ with abundant vitamin C and phenolic substances, which can enhance both the flavour and the nutrition of the CFJ. Calcium ions are necessary for the LMP to produce jelly [38], similar to the results of previously published studies [39], pectin can form relatively good gels and be suitable for 3D food printing at a calcium ion concentration of 1.4 mg/mL. The main objective of RSM is to obtain the optimum formulation of the functional ingredients [40]. In our experiments, the optimized CFJ sensory acceptability was high, with significant effects of orange juice and pectin on the overall sensory evaluation.

Nowadays, the typical way to produce large-scale jellies is to use traditional plastic moulds, but the limitations of the moulds make it challenging to produce more complex shapes [41]. Compared with traditional tactile tools, 3D food printing is more capable of meeting the needs of consumers for personalization [42]. The most successful examples of 3D printing applications in the food industry are chocolates [43], in addition to similar paste-like foods, such as mashed potatoes [44] and fruit purees [29]. As a result of the test, the CFJ made using the optimal formula can meet the requirements of 3D food printing and successfully print the desired food shape. Although the cost of using traditional moulds, to obtain jelly shapes, is lower at this stage, many people are unsatisfied with the same old food shapes. Thus, personalization will be the future trend, and it is necessary to explore the 3D food printing of the CFJ.

Similarly, Khouryieh, H.A. et al. prepared a low-calorie grape jelly containing 10 calories per serving [45]. Acosta O. et al. produced a low-calorie blackberry jelly that offered under 8 calories per recommended amount [46] and a mixed fruit jelly that offered less than 12 calories for each recommended amount [47]. Previous studies reported that foods with reference intakes defined as 30 g or less and not exceeding 40 calories per reference serving, were considered low-calorie products [47]. Actually, based on the comprehensive consideration of the recommended intake of each ingredient in the CFJ, the daily intake of the CFJ should be less than 30 g, thus providing less than 25.8 calories per serving of the CFJ.

The results showed that the CFJ is rich in phenols and flavonoids (12.44 ± 0.26 GAE mg/g and 3.64 ± 0.21 RE mg/g fresh weight, respectively), which may be due to the inclusion of *chenpi* and orange juice. Both *chenpi* [20] and orange juice [48] are rich in phenolic compounds, and flavonoids are the most abundant phenols [49]. The total phenol content (TPC) in the dried peels of *Citrus reticulata* (*chenpi*) was about 42–51.8 mg GAE/g, and the total flavonoid content (TFC) was approximately14.0–31.9 mg/g [17], which varies from the place of origin. Moreover, orange juice’s total phenols and flavonoids varied significantly with different varieties and sources. The TPC of 100% orange juice was 34 mg GAE/500 mL, determined by Dourado, G.K. et al. [50], and the TPC and TFC in fresh orange juice were about 89.0 ± 5.4 mg GAE/100 mL and 57.3 mg RE/100 mL, determined by Vieira F.N. et al. [51].

The ABTS, DPPH, and FRAP methods are commonly performed to determine the antioxidant capacity in vitro [52]. Since the reaction principles of the three assays are different, it is necessary to apply them jointly to obtain a better precise result for the antioxidant ability [53,54]. Citrus fruits are generally considered to have the good antioxidant capacity. Studies by Chunhua Zhu et al. indicated that the ABTS scavenging ability of citrus varied from 72.94 to 45.37 mmol TE/g fresh weight (FW), 10.29 to 21.31 mmol TE/g FW, in the DPPH assay, and 10.8 to 17.66 mmol TE/g FW, in the FRAP assay [55]. Kopjar, M. et al. developed a gel with citrus fiber/blackberry and its antioxidant ability in ABTS, DPPH, and FRAP assays were 1.526 ± 0.099 µmol TE/100 g, 216.55 ± 1.57 nmol TE/100 g, and 291.05 ± 0.96 nmol TE/100 g, respectively [56]. A significantly higher scavenging of ABTS by the CFJ than by the DPPH assay and the FRAP test was observed in our study. This difference may be due to the fact that the various antioxidants present in the CFJ respond in a different manner to the free radicals or ferric ions used in the test method [52]. Furthermore, the scavenging capacity of the CFJ for ABTS was remarkably higher than that of DPPH, due to the ABTS test tending to react with highly pigmented and hydrophilic antioxidants, more than with the DPPH method [57]. Similar findings were reported in previous studies [33], where the antioxidant ability of fruit or vegetable extracts, determined by the ABTS method, was better than with the DPPH and FRAP methods [57]. Additionally, the vitamin C in orange juice is extremely susceptible to oxidation by oxygen in the air [58,59]. Although we have tried to minimize the time of exposure to air when preparing and measuring the antioxidant activity of the jelly, such as measuring the antioxidant activity immediately after the sample preparation, some of the vitamin C in the jelly will still be oxidized. Therefore, the actual antioxidant activity of the jelly may be larger than the experimentally measured value. We expect to minimize the oxidation of vitamin C during further production of the jelly, in the future.

We noticed a decline in body weight gain and white adipose tissue (WAT) and an increase in brown adipose tissue (BAT) in healthy mice, after the consumption of high concentrations of the CFJ. The primary role of WAT is to store energy, while BAT is responsible for thermogenesis [60]. Our results suggest that the CFJ may exert an anti-obesity effect by promoting thermogenesis in the body. Meanwhile, the anti-obesity effect of the CFJ may be closely related to *chenpi* [61]. The *Chenpi* extract was reported to reduce the adipogenesis of 3T3-L1 adipocytes [62] and alleviate obesity and hepatic steatosis induced by a high-fat diet [63]. Moreover, orange juice, another major component of the CFJ, may have a significant role, due to its richness in vitamin C and flavonoids. Moro (a blood orange) juice was reported to inhibit body weight gain, inguinal fat accumulation [64], and fatty liver in high-fat diet mice [65]. Furthermore, a clinical trial has shown that orange juice can reduce weight and improve obesity-related symptoms when combined with a reduced-calorie diet [66]. In general, the mechanisms by which dietary fiber contributes to weight loss include delaying gastric emptying, reducing glucose diffusion, and preventing fat absorption [67]. Dietary fibers that can form gels, similar to pectin, could bind bound bile acids and micelle components [68], which in turn reduce the fat absorption in the intestine. It may be key to the pectin-mediated reductions in weight gain. Moreover, Pectin is a kind of soluble dietary fiber that could improve obesity and the metabolic syndrome, and increase satiety after intake [69]. Therefore, pectin could reduce weight by increasing satiety [70]. In our experiments, the mice’s body weight, food intake and liver weight were reduced after high concentrations of the CFJ were administered. It is indicated that the anti-obesity effect of the CFJ may be achieved by increasing the satiety in mice.

Generally, the high levels of TC, TG, LDL-C and the low levels of HDL-C in the serum are positively correlated with obesity and hyperlipidemia [71]. A study by Qian Y. et al. has shown that the administration of *chenpi* powder reduced the TC concentration in healthy mice [20]. In a similar study, serum HDL-C levels in healthy mice were significantly reduced after supplementation with low concentrations of Ganpu tea [72]. In our experiment, it was observed that the serum TC, TG, and LDL-C levels of mice supplemented with the CFJ were obviously higher than those of the C group. At the same time, the HDL-C concentration was markedly lower than that of the C group. However, this phenomenon was alleviated with the increase in the supplementary concentration of the CFJ. It may be related to the appetite and body weight of mice. The appetite of mice was slightly increased by the intake of the CFJ at low concentrations, resulting in the higher body weight of mice in group L, than in group C (but not significantly). However, with the increase in the CFJ concentration, the appetite of the mice was inhibited and their body weight decreased.

CAT and SOD are necessary antioxidant enzymes in the body, and their synergistic effect can scavenge free radicals [73]. Our experiment has shown that supplementing the CFJ with medium and high concentrations could enhance the antioxidant capacity in vivo by increasing the CAT and SOD activity. It can be explained by the *chenpi* decoction. *Chenpi* is rich in poly methoxy flavones (PMFs) [74], which may provide anti-inflammatory and antioxidant benefits [17]. Furthermore, these antioxidant effects may be partly associated with the concentration of vitamin C in orange juice. This result corresponds to the in vitro antioxidant assay of the CFJ. Overall, the potential anti-obesity and antioxidants exhibited by the CFJ in healthy mice may result from the combined effect of several main components.

## 5. Conclusions

In summary, a kind of formulation of the functional jelly was developed, based on citrus products, including *chenpi*, orange juice, and citrus pectin, in this study. Our results show that the CFJ we developed is a healthy and beneficial snack rich in bioactive substances with low-calorie and antioxidant in vitro properties. The CFJ prepared by the best formula could also be personalized by the 3D food printing technology for the jelly shape. Moreover, in vivo results show that supplementing the CFJ daily may prevent obesity and improve the antioxidant ability. The development of the CFJ in this study used citrus-based raw materials, which could promote the utilization of citrus by-products and the healthy development of the citrus processing industry.

## Figures and Tables

**Table 1:** The evaluation criterion of the CFJ.

Indicators	Scoring Details
Colour and lustre (10%)	Dark brown and well-distributed in colour (7–10%). Brown and evenly coloured (4–6%). Light brown and non-uniform in colour (0–3%).
Texture (30%)	Superior chewiness, uniform texture without bubbles, delicate, and smooth (21–30%). Nice chewiness, the texture is basically uniform, with a few bubbles (11–20%). Poor toughness, non-uniform texture, with a large amount of bubbles (0–10%).
Aroma (30%)	Rich aroma of *chenpi* and orange flavour with no bad odour (21–30%). Slight aroma of *chenpi* and orange flavour (11–20%). Does not have an obvious aroma of *chenpi* and orange, and has an undesirable flavour (0–10%).
Taste (30%)	A smooth and delicate taste, with the best sweet and sour taste, and no noticeable post-bitterness (21–30%). Delicate taste in general, with a suitably sweet and sour taste, and accompanied by a post-bitter or mild acidity (11–20%). Tastes rough, too sour or too sweet, with a heavy bitterness (0–10%).

**Table 2:** Variables and the range of values for the SFE and RSM.

Factors of the SFE	Variables					
Addition of *chenpi* decoction (%)	10	15	20	25	30	35
Addition of orange juice (%)	15	20	25	30	35	40
Addition of pectin (%)	1	2	3	4	5	6
Concentration of CaCL_2_ (mg/mL)	0.8	1.0	1.2	1.4	1.6	1.8
**Independent Variables of the RSM**	**Levels**					
	**−1**		**0**		**1**	
A: addition of *chenpi* decoction (%)	25		30		35	
B: addition of orange juice (%)	20		25		30	
C: addition of pectin (%)	3		4		5	

**Table 3:** The main nutritional composition of a normal diet.

Ingredients	Crude Protein	Ether Extract	Crude Fiber	Crude Ash Powder	Water Content	Calcium	Phosphorus
Content	>18%	>4%	<5%	<8%	<10%	1.0–1.8%	0.6–1.2%

**Table 4:** Experimental design and the results of the CFJ response surface design.

Run	A: *Chenpi* Decoction (%)	B: Orange Juice (%)	C: Pectin (%)	R: Total Sensory Evaluation Score
1	35	25	5	60.12 ± 3.37
2	35	20	4	71.16 ± 2.35
3	30	25	4	83.30 ± 3.56
4	35	30	4	64.20 ± 4.89
5	25	25	3	69.15 ± 4.04
6	35	25	3	74.10 ± 3.60
7	25	25	5	67.90 ± 4.08
8	30	25	4	83.80 ± 2.62
9	30	25	4	82.00 ± 3.73
10	30	25	4	81.30 ± 3.11
11	30	30	5	66.20 ± 3.17
12	30	20	5	80.90 ± 2.02
13	30	20	3	88.19 ± 2.03
14	25	20	4	83.00 ± 4.10
15	30	25	4	81.00 ± 3.94
16	25	30	4	63.90 ± 3.87
17	30	30	3	71.10 ± 4.38
18 ^1^	29.12	20	3.61	89.16 ± 2.13

^1^ The optimal formula and its predicted values of R, based on the regression equation.

**Table 5:** Analysis of variance results for the CFJ ^1^.

Source	Sum of Squares	df	Mean Square	F-Value	*p*-Value	
Model ^1^	1176.98	9	130.78	40.6	<0.0001	significant
A-*Chenpi* decoction	28.13	1	28.13	8.73	0.0213	
B-Orange juice	420.5	1	420.5	130.53	<0.0001	
C-Pectin	91.13	1	91.13	28.29	0.0011	
AB	36	1	36	11.18	0.0124	
AC	42.25	1	42.25	13.12	0.0085	
BC	1	1	1	0.3104	0.5948	
A ²	440.21	1	440.21	136.65	<0.0001	
B ²	9.16	1	9.16	2.84	0.1356	
C ²	75.16	1	75.16	23.33	0.0019	
Residual	22.55	7	3.22			
Lack of Fit	15.75	3	5.25	3.09	0.1523	not significant

^1^ R² = 0.9812, Adj. R² = 0.957, C.V. (%) = 2.4, adequate precision = 20.61.

**Table 6:** Nutritional composition of the optimised citrus-based functional jelly.

Composition	Per 100 g Fresh Weight	NRV% ^1^
energy	360 ± 4.35 (kJ)	4%
protein	0.53 ± 0.01 (g)	1%
fat	0.1 ± 0.02 (g)	0%
carbohydrate	20.4 ± 0.45 (g)	7%
sodium	18.3 ± 0.32 (mg)	1%

^1^ Nutrient reference values (NRVs) refer to the abbreviation of the “Chinese food label nutrient reference values”, which is a reference standard dedicated to food labelling, comparing the amount of the nutrient content of the food, and is a nutritional reference scale for consumers when choosing food. Nutrient reference values are mainly based on the recommended daily intake (RNI) and the appropriate intake (AI) of the dietary nutrients for Chinese residents.

**Table 7:** The concentration of the specific phenols and flavonoids contained in the CFJ.

Components	Content (µg/g Fresh Weight)	Components	Content (µg/g Fresh Weight)
Hesperidin	509.71 ± 1.60	Tangeretin	1.79 ± 0.13
Nobiletin	143.10 ± 3.21	Luteolin	1.09 ± 0.16
Naringin	116.68 ± 1.53	Chrysin	0.91 ± 0.06
Diosmin	17.78 ± 0.33	L-epicatechin	0.82 ± 0.19
Naringenin	15.21 ± 0.62	Baicalin	0.54 ± 0.17
Sinensetin	15.20 ± 0.37	Fisetin	0.38 ± 0.04
Vitexin	11.38 ± 0.29	Cynaroside	0.25 ± 0.02
Rutin	8.94 ± 0.18	Taxifolin	0.230.00
Neohesperidin	8.10 ± 0.25	Catechin	0.22 ± 0.01
Isovitexin	6.66 ± 0.22	Quercetin	0.14 ± 0.02
Apigenin	3.25 ± 0.19	Kaempferide	0.11 ± 0.02
Puerarin	2.13 ± 0.09	Quercitrin	0.03 ± 0.01

## Data Availability

Data are contained within the article.

## References

[1] Cani P.D. (2019). Microbiota and metabolites in metabolic diseases. Nat. Rev. Endocrinol..

[2] Garcia-Rios A., Torres-Pena J.D., Perez-Jimenez F., Perez-Martinez P. (2017). Gut Microbiota: A New Marker of Cardiovascular Disease. Curr. Pharm. Des..

[3] Farooqui A.A. (2015). Effect of Long Term Consumption of High Calorie Diet and Calorie Restriction on Human Health. High Calorie Diet and the Human Brain.

[4] Sun-Waterhouse D. (2011). The development of fruit-based functional foods targeting the health and wellness market: A review. Int. J. Food Sci. Technol..

[5] Hosseini B., Saedisomeolia A., Allman-Farinelli M. (2017). Association Between Antioxidant Intake/Status and Obesity: A Systematic Review of Observational Studies. Biol. Trace Elem. Res..

[6] Terra X., Montagut G., Bustos M., Llopiz N., Ardevol A., Blade C., Fernandez-Larrea J., Pujadas G., Salvado J., Arola L. (2009). Grape-seed procyanidins prevent low-grade inflammation by modulating cytokine expression in rats fed a high-fat diet. J. Nutr. Biochem..

[7] Fernandez-Sanchez A., Madrigal-Santillan E., Bautista M., Esquivel-Soto J., Morales-Gonzalez A., Esquivel-Chirino C., Durante-Montiel I., Sanchez-Rivera G., Valadez-Vega C., Morales-Gonzalez J.A. (2011). Inflammation, oxidative stress, and obesity. Int. J. Mol. Sci..

[8] Anigboro A.A., Avwioroko O.J., Akeghware O., Tonukari N.J. (2021). Anti-obesity, antioxidant and in silico evaluation of Justicia carnea bioactive compounds as potential inhibitors of an enzyme linked with obesity: Insights from kinetics, semi-empirical quantum mechanics and molecular docking analysis. Biophys. Chem..

[9] Puchau B., Ochoa M.C., Zulet M.A., Marti A., Martinez J.A., Members G. (2010). Dietary total antioxidant capacity and obesity in children and adolescents. Int. J. Food Sci. Nutr..

[10] Muller M., Canfora E.E., Blaak E.E. (2018). Gastrointestinal Transit Time, Glucose Homeostasis and Metabolic Health: Modulation by Dietary Fibers. Nutrients.

[11] Nie Q., Chen H., Hu J., Fan S., Nie S. (2019). Dietary compounds and traditional Chinese medicine ameliorate type 2 diabetes by modulating gut microbiota. Crit. Rev. Food Sci. Nutr..

[12] Simpson E.J., Mendis B., Macdonald I.A. (2016). Orange juice consumption and its effect on blood lipid profile and indices of the metabolic syndrome; a randomised, controlled trial in an at-risk population. Food Funct..

[13] Kelebek H., Selli S. (2011). Determination of volatile, phenolic, organic acid and sugar components in a Turkish cv. Dortyol (*Citrus sinensis* L. Osbeck) orange juice. J. Sci. Food Agric..

[14] Kawser Hossain M., Abdal Dayem A., Han J., Yin Y., Kim K., Kumar Saha S., Yang G.M., Choi H.Y., Cho S.G. (2016). Molecular Mechanisms of the Anti-Obesity and Anti-Diabetic Properties of Flavonoids. Int. J. Mol. Sci..

[15] Yu X., Sun S., Guo Y., Liu Y., Yang D., Li G., Lu S. (2018). Citri Reticulatae Pericarpium (Chenpi): Botany, ethnopharmacology, phytochemistry, and pharmacology of a frequently used traditional Chinese medicine. J. Ethnopharmacol..

[16] Li X.-T., Zhao J.A. (2012). An approach to the nature of Qi in TCM-Qi and bioenergy. Recent Advances in Theories and Practice of Chinese Medicine.

[17] Chen X.M., Tait A.R., Kitts D.D. (2017). Flavonoid composition of orange peel and its association with antioxidant and anti-inflammatory activities. Food Chem..

[18] Hu M., Zhang L., Ruan Z., Han P., Yu Y. (2021). The Regulatory Effects of Citrus Peel Powder on Liver Metabolites and Gut Flora in Mice with Non-Alcoholic Fatty Liver Disease (NAFLD). Foods.

[19] Ashraf H., Butt M.S., Iqbal M.J., Suleria H.A.R. (2017). Citrus peel extract and powder attenuate hypercholesterolemia and hyperglycemia using rodent experimental modeling. Asian Pac. J. Trop. Biomed..

[20] Qian Y., Gao Z., Wang C., Ma J., Li G., Fu F., Guo J., Shan Y. (2021). Effects of Different Treatment Methods of Dried Citrus Peel (Chenpi) on Intestinal Microflora and Short-Chain Fatty Acids in Healthy Mice. Front. Nutr..

[21] Beukema M., Faas M.M., de Vos P. (2020). The effects of different dietary fiber pectin structures on the gastrointestinal immune barrier: Impact via gut microbiota and direct effects on immune cells. Exp. Mol. Med..

[22] Blanco-Perez F., Steigerwald H., Schulke S., Vieths S., Toda M., Scheurer S. (2021). The Dietary Fiber Pectin: Health Benefits and Potential for the Treatment of Allergies by Modulation of Gut Microbiota. Curr. Allergy Asthma Rep..

[23] Wang Y.T., Lien L.L., Chang Y.C., Wu J.S. (2013). Pectin methyl esterase treatment on high-methoxy pectin for making fruit jam with reduced sugar content. J. Sci. Food Agric..

[24] Nuramalia D.R., Damayanthi E. (2018). Effect of green okra and strawberry ratio on antioxidant activity, total phenolic content, and organoleptic properties of jelly drink. IOP Conf. Ser. Earth Environ. Sci..

[25] Pereira P.A.P., Santos O.D.H.d., Monteiro R.d.S., Jerusa Josiane Francisca de Jesus Lima F.M.D., Lima M.B. (2019). Characterization and influence of hydrocolloids on low caloric orange jellies. Emir. J. Food Agric..

[26] Duan H., Wang X., Azarakhsh N., Wang C., Li M., Fu G., Huang X. (2022). Optimization of calcium pectinate gel production from high methoxyl pectin. J. Sci. Food Agric..

[27] Peng M., Gao Z., Liao Y., Guo J., Shan Y. (2022). Development of Functional Kiwifruit Jelly with chenpi (FKJ) by 3D Food Printing Technology and Its Anti-Obesity and Antioxidant Potentials. Foods.

[28] Yolmeh M., Jafari S.M. (2017). Applications of Response Surface Methodology in the Food Industry Processes. Food Bioprocess Technol..

[29] Derossi A., Caporizzi R., Azzollini D., Severini C. (2018). Application of 3D printing for customized food. A case on the development of a fruit-based snack for children. J. Food Eng..

[30] Li X., Chen D., Mai Y., Wen B., Wang X. (2012). Concordance between antioxidant activities in vitro and chemical components of Radix Astragali (Huangqi). Nat. Prod. Res..

[31] Yamin R., Mistriyani S., Ihsan S., Armadany F.I., Sahumena M.H., Fatimah W.O.N. (2020). Determination of total phenolic and flavonoid contents of jackfruit peel and in vitro antiradical test. Food Res..

[32] Formagio A.S., Volobuff C.R., Santiago M., Cardoso C.A., Vieira Mdo C., Valdevina Pereira Z. (2014). Evaluation of Antioxidant Activity, Total Flavonoids, Tannins and Phenolic Compounds in Psychotria Leaf Extracts. Antioxidants.

[33] Thaipong K., Boonprakob U., Crosby K., Cisneros-Zevallos L., Hawkins Byrne D. (2006). Comparison of ABTS, DPPH, FRAP, and ORAC assays for estimating antioxidant activity from guava fruit extracts. J. Food Compos. Anal..

[34] Wang P., Li D., Ke W., Liang D., Hu X., Chen F. (2020). Resveratrol-induced gut microbiota reduces obesity in high-fat diet-fed mice. Int. J. Obes..

[35] Ke Z., Zhao Z., Zhao Y., Xu X., Li Y., Tan S., Huang C., Zhou Z. (2018). PMFs-rich Citrus extract prevents the development of non-alcoholic fatty liver disease in C57BL/6J mice induced by a high-fat diet. J. Funct. Foods.

[36] Tang X., Zhao H., Jiang W., Zhang S., Guo S., Gao X., Yang P., Shi L., Liu L. (2018). Pharmacokinetics and pharmacodynamics of citrus peel extract in lipopolysaccharide-induced acute lung injury combined with Pinelliae Rhizoma Praeparatum. Food Funct..

[37] Liu N., Sun J., Yang W., Liang D., Guo L., Li X., Gao W. (2022). Evaluation of bioactive flavonoids in Citri Reticulatae Pericarpium from different regions and its association with antioxidant and α-glucosidase inhibitory activities. Tradit. Med. Res..

[38] Fraeye I., Duvetter T., Doungla E., Van Loey A., Hendrickx M. (2010). Fine-tuning the properties of pectin–calcium gels by control of pectin fine structure, gel composition and environmental conditions. Trends Food Sci. Technol..

[39] Vancauwenberghe V., Katalagarianakis L., Wang Z., Meerts M., Hertog M., Verboven P., Moldenaers P., Hendrickx M.E., Lammertyn J., Nicolaï B. (2017). Pectin based food-ink formulations for 3-D printing of customizable porous food simulants. Innov. Food Sci. Emerg..

[40] Zhang L., Zeng Y., Cheng Z. (2016). Removal of heavy metal ions using chitosan and modified chitosan: A review. J. Mol. Liq..

[41] Darade A.D., Mundada A.S. (2021). Oral medicated jellies as a emerging platform for oral drug delivery in pediatrics. World J. Pharm. Res..

[42] Lee B., Hong J., Surh J., Saakes D. Ori-mandu: Korean Dumpling into Whatever Shape You Want. Proceedings of the 2017 Conference on Designing Interactive Systems.

[43] Khemacheevakul K., Wolodko J., Nguyen H., Wismer W. (2021). Temporal Sensory Perceptions of Sugar-Reduced 3D Printed Chocolates. Foods.

[44] Liu Z., Bhandari B., Prakash S., Zhang M. (2018). Creation of internal structure of mashed potato construct by 3D printing and its textural properties. Food Res. Int..

[45] Khouryieh H.A., Aramouni F.M., Herald T.J. (2005). Physical, chemical and sensory properties of sugar-free jelly. J. Food Qual..

[46] Acosta O., Víquez F., Cubero E., Morales I. (2006). Ingredient Levels Optimization and Nutritional Evaluation of a Low-calorie Blackberry (*Rubus irasuensis* Liebm.) Jelly. J. Food Sci..

[47] Acosta O., Víquez F., Cubero E. (2008). Optimisation of low calorie mixed fruit jelly by response surface methodology. Food Qual. Prefer..

[48] Mesquita E., Monteiro M. (2018). Simultaneous HPLC determination of flavonoids and phenolic acids profile in Pera-Rio orange juice. Food Res. Int..

[49] Zhu M., Huang Y., Wang Y., Shi T., Zhang L., Chen Y., Xie M. (2019). Comparison of (poly)phenolic compounds and antioxidant properties of pomace extracts from kiwi and grape juice. Food Chem..

[50] Dourado G.K., Cesar T.B. (2015). Investigation of cytokines, oxidative stress, metabolic, and inflammatory biomarkers after orange juice consumption by normal and overweight subjects. Food Nutr. Res..

[51] Vieira F.N., Lourenco S., Fidalgo L.G., Santos S.A.O., Silvestre A.J.D., Jeronimo E., Saraiva J.A. (2018). Long-Term Effect on Bioactive Components and Antioxidant Activity of Thermal and High-Pressure Pasteurization of Orange Juice. Molecules.

[52] Shah P., Modi H.A. (2015). Comparative Study of DPPH, ABTS and FRAP Assays for Determination of Antioxidant Activity. Int. J. Res. Appl. Sci. Eng. Technol..

[53] Kim D.O., Lee K.W., Lee H.J., Lee C.Y. (2002). Vitamin C Equivalent Antioxidant Capacity (VCEAC) of Phenolic Phytochemicals. J. Agric. Food Chem..

[54] Müller L., Fröhlich K., Böhm V. (2011). Comparative antioxidant activities of carotenoids measured by ferric reducing antioxidant power (FRAP), ABTS bleaching assay (αTEAC), DPPH assay and peroxyl radical scavenging assay. Food Chem..

[55] Zhu C., Zhou X., Long C., Du Y., Li J., Yue J., Pan S. (2020). Variations of Flavonoid Composition and Antioxidant Properties among Different Cultivars, Fruit Tissues and Developmental Stages of Citrus Fruits. Chem. Biodivers..

[56] Kopjar M., Ivic I., Buljeta I., Corkovic I., Vukoja J., Simunovic J., Pichler A. (2021). Volatiles and Antioxidant Activity of Citrus Fiber/Blackberry Gels: Influence of Sucrose and Trehalose. Plants.

[57] Floegel A., Kim D.-O., Chung S.-J., Koo S.I., Chun O.K. (2011). Comparison of ABTS/DPPH assays to measure antioxidant capacity in popular antioxidant-rich US foods. J. Food Compos. Anal..

[58] Mditshwa A., Magwaza L.S., Tesfay S.Z., Opara U.L. (2017). Postharvest factors affecting vitamin C content of citrus fruits: A review. Sci. Horticult..

[59] Van Bree I., Baetens J.M., Samapundo S., Devlieghere F., Laleman R., Vandekinderen I., Noseda B., Xhaferi R., De Baets B., De Meulenaer B. (2012). Modelling the degradation kinetics of vitamin C in fruit juice in relation to the initial headspace oxygen concentration. Food Chem..

[60] Alcala M., Calderon-Dominguez M., Serra D., Herrero L., Viana M. (2019). Mechanisms of Impaired Brown Adipose Tissue Recruitment in Obesity. Front. Physiol..

[61] Liu D., Chen C., Li R. (2011). Protective effect of flavonoids from pericarpium citri reticulatae (chenpi) against oxidative stress induced by exhaustive exercise. Afr. J. Microbiol. Res..

[62] Guo J., Cao Y., Ho C.-T., Jin S., Huang Q. (2017). Aged citrus peel (chenpi) extract reduces lipogenesis in differentiating 3T3-L1 adipocytes. J. Funct. Foods.

[63] Falduto M., Smedile F., Zhang M., Zheng T., Zhu J., Huang Q., Weeks R., Ermakov A.M., Chikindas M.L. (2022). Anti-obesity effects of Chenpi: An artificial gastrointestinal system study. Microb. Biotechnol..

[64] Titta L., Trinei M., Stendardo M., Berniakovich I., Petroni K., Tonelli C., Riso P., Porrini M., Minucci S., Pelicci P.G. (2010). Blood orange juice inhibits fat accumulation in mice. Int. J. Obes..

[65] Salamone F., Li Volti G., Titta L., Puzzo L., Barbagallo I., La Delia F., Zelber-Sagi S., Malaguarnera M., Pelicci P.G., Giorgio M. (2012). Moro orange juice prevents fatty liver in mice. World J. Gastroenterol..

[66] Ribeiro C., Dourado G., Cesar T. (2017). Orange juice allied to a reduced-calorie diet results in weight loss and ameliorates obesity-related biomarkers: A randomized controlled trial. Nutrition.

[67] Bray J.K., Chiu G.S., McNeil L.K., Moon M.L., Wall R., Towers A.E., Freund G.G. (2018). Switching from a high-fat cellulose diet to a high-fat pectin diet reverses certain obesity-related morbidities. Nutr. Metab..

[68] Kaczmarczyk M.M., Miller M.J., Freund G.G. (2012). The health benefits of dietary fiber: Beyond the usual suspects of type 2 diabetes mellitus, cardiovascular disease and colon cancer. Metabolism.

[69] Elshahed M.S., Miron A., Aprotosoaie A.C., Farag M.A. (2021). Pectin in diet: Interactions with the human microbiome, role in gut homeostasis, and nutrient-drug interactions. Carbohydr. Polym..

[70] Adam C.L., Thomson L.M., Williams P.A., Ross A.W. (2015). Soluble Fermentable Dietary Fibre (Pectin) Decreases Caloric Intake, Adiposity and Lipidaemia in High-Fat Diet-Induced Obese Rats. PLoS ONE.

[71] Stepien A., Stepien M., Wlazel R.N., Paradowski M., Banach M., Rysz J. (2014). Assessment of the relationship between lipid parameters and obesity indices in non-diabetic obese patients: A preliminary report. Med. Sci. Monit..

[72] Wang C., Gao Z., Qian Y., Li X., Wang J., Ma J., Guo J., Fu F. (2021). Effects of Different Concentrations of Ganpu Tea on Fecal Microbiota and Short Chain Fatty Acids in Mice. Nutrients.

[73] Huang Q.H., Xu L.Q., Liu Y.H., Wu J.Z., Wu X., Lai X.P., Li Y.C., Su Z.R., Chen J.N., Xie Y.L. (2017). Polydatin Protects Rat Liver against Ethanol-Induced Injury: Involvement of CYP2E1/ROS/Nrf2 and TLR4/NF-kappaB p65 Pathway. Evid.-Based Complement. Altern. Med..

[74] Yang M., Jiang Z., Wen M., Wu Z., Zha M., Xu W., Zhang L. (2022). Chemical Variation of Chenpi (Citrus Peels) and Corresponding Correlated Bioactive Compounds by LC-MS Metabolomics and Multibioassay Analysis. Front. Nutr..

